# The prevalence of dental erosion in Nigerian patients with gastro-oesophageal reflux disease

**DOI:** 10.1186/1472-6831-5-1

**Published:** 2005-03-01

**Authors:** Adeleke O Oginni, Elugwaraonu A Agbakwuru, Dennis A Ndububa

**Affiliations:** 1 Department of Restorative Dentistry, College of Health Sciences, Obafemi Awolowo University, Ile-Ife, Nigeria; 2 Department of Surgery, College of Health Sciences, Obafemi Awolowo University, Ile-Ife, Nigeria; 3 Department of Medicine, College of Health Sciences, Obafemi Awolowo University, Ile-Ife, Nigeria

## Abstract

**Background:**

In various people of the Western world, gastro-oesophageal reflux (GOR) has been reported to be a common problem. Various studies have also assessed the relationship between GOR and dental erosion. The authors are not aware of such studies in Nigerians. It is therefore the aims of the present study to estimate the prevalence of GOR; to estimate the prevalence of dental erosion in patients with GORD; to document the oral findings in patients diagnosed with GORD and to compare these findings with previous studies elsewhere.

**Methods:**

A total of 225 subjects comprising of 100 volunteers and 125 patients diagnosed with GORD were involved in this study. History of gastric juice regurgitation and heartburn were recorded. Oral examination to quantify loss of tooth structure was done using the tooth wear index (TWI) designed by Smith and Knight (1984).

**Results:**

Twenty patients with GORD presented with dental erosion in the maxillary anterior teeth with TWI scores ranging from 1–3. The prevalence of erosion was found to be statistically significant between GORD patients (16%) and control (5%) (p < 0.05), but not significant between endoscopic diagnostic groups (p > 0.05).

**Conclusion:**

The present study supports the consideration of dental erosion as the extra-oesophageal manifestation of GORD. However the association between GORD and burning mouth sensation needs more investigation.

## Background

Gastro-oesophageal reflux (GOR) is the passage of gastric contents into the oesophagus. Once past the upper oesophageal sphincter, the gastric juice may pass into the oral cavity. The continual exposure of the teeth and other oral structures to gastric refluxate may result in dental erosion and other soft tissue symptoms [1]. Any acid with a pH below the critical pH of dental enamel dissolution (5.5) can dissolve the hydroxyapatite crystals in enamel. However the critical pH below which enamel dissolves is not constant but is rather inversely proportional to the concentrations of calcium and phosphate in the saliva and plaque fluid [2]. Gastric refluxate has a pH of less than 2.0 and thus has the potential to cause dental erosion [3]. Acid regurgitation is a common symptom of upper gastro-intestinal tract disorders and dysfunctions such as peptic ulcer (duodenal and gastric ulcers) and reflux oesophagitis [4]. Other symptoms include heartburn, non-cardiac epigastric and retrosternal pain [5]. Prominent among factors precipitating GOR and its complications, gastro-oesophageal reflux disease (GORD) are fatty diets and alcohol.

Current understanding of GOR and GORD is that acid reflux into the oesophagus may be caused by three possible mechanisms [6]:

(1) Transient spontaneous or inappropriate relaxations of the sphincter;

(2) Transient increase in intra-abdominal or intragastric pressure;

(3) Functional abnormality of the lower oesophageal sphincter (LOS).

Bargen and Austin in 1937 [7] first reported the link between dental erosion and gastro-intestinal disturbances. Since then there has been several other studies confirming the relationship between this loss of tooth structure and GORD in the United Kingdom (UK) [8], United States of America (U.S.A) [9] and Canada [10]. A recent survey among young people in the U.K also revealed an association between dental erosion and symptoms of GOR [11]. Although soft tissue symptoms (non-specific burning sensation in the mouth) have been mentioned in the literature, pathognomonic soft tissue lesions have not been documented [1]. But some researchers have reported a lack of relationship between periodontal lesions and GOR, since the prevalence of periodontal lesions is similar in patients with GORD and in healthy volunteers [12]. In various people of the Western world, GOR has been reported to be a common problem, often related to meals and occurring in about 60% of the population at some point in their lives [13]. Whereas the prevalence of GORD is estimated to range from 6% – 10% [14,15], Meurman *et al *[16] examined 117 patients with GORD, of whom 28 (24%) had dental erosion. Also, Schroeder *et al *[17] identified dental erosion in 11 (55%) of 20 patients with GORD. The authors are not aware of such figures in Nigerians. A search of the literature also revealed a dearth of information on this condition and its complications among Black Africans. It is therefore the aim of the present study to estimate the prevalence of GOR among patients attending the medical out-patient department of the Obafemi Awolowo University Teaching Hospital Complex Ile-Ife, Nigeria; to estimate the prevalence of dental erosion in patients with GORD; to document the oral findings in patients diagnosed with GORD and to compare these findings with previous studies elsewhere.

## Methods

A total of 225 subjects were involved in the study over a period of 6 months, January – June 2002. The subjects were made up of 100 volunteers attending the medical outpatient department (MOPD) and 125 patients diagnosed with gastro-oesophageal reflux disease among whom the prevalence of GOR and GORD was determined respectively. Their age ranges from 18 – 72 years, with a mean age ± S.D. of 38 ± 10.87 years. Patients presenting primarily with symptoms of asthma, bronchitis and other respiratory disorders were excluded, since they are also at risk of dental erosion [18].

To assess the prevalence of GOR and GORD, consenting patients attending the medical outpatient department of the Obafemi Awolowo University Teaching Hospital's complex Ile-Ife, Nigeria, were questioned with reference to their experience regarding symptoms of GOR such as regurgitation of gastric juice, epigastric and non-cardiac pain (heartburn). The onset, frequency of occurrence and duration of each of the symptoms were ascertained and recorded. Patients presenting with a history of heartburn 2 or more times per week were diagnosed as having GORD^10^. Oral examination was carried out to quantify any loss of tooth structure using the tooth wear index (TWI) designed by Smith and Knight (1984) [19].

To document the oral findings associated with GORD, patients referred to the Gastro-intestinal (GIT) endoscopic unit for investigation of gastro-oesophageal tract disease were also evaluated for dental erosion and other soft tissue symptoms such as non-specific burning sensation in the mouth and sensitivity in the tongue. The dental evaluation included history to determine potential eatiological factors responsible for dental erosion. Patients were also examined clinically to quantify loss of tooth structure using the tooth wear index. The oral examination was performed by one of the authors (AO), blind as to the endoscopic diagnosis of subjects. Patients with positive endoscopic findings plus the occurrence of heartburn two or more times per-week were assessed to meet the criteria for GORD. When a clear-cut endoscopic evidence of oesophageal inflammation was seen (marked redness, fibrinous membrane, and or ulcerations), a diagnosis of oesophagitis was made. Gastroduodenal ulcer was diagnosed through endoscopic evidence of an ulcer with a necrotic base or a clear-cut scar. This was grouped into those with gastric ulcer (ulcer in the pylorus, antrum, corpus or fundus) and those with duodenal ulcer (ulcer in the bulbus). Only one diagnosis was given for each patient.

Data collected were entered into a computer and analysed using chi-square. P value <0.05 were considered significant.

## Results

Thirty-five of the 100 subjects attending the medical outpatient department had acid regurgitation and acidic taste sensation in the mouth. This occurs most of the time following a full stomach meal and is associated with belching. Table 1 shows the prevalence of GOR and GORD. Eleven reported a history of heartburn 2 or more times per week, while 16 and 10 reported weekly and monthly history of heartburn respectively. Comparison of subjects presenting with heartburn 2 or more times per week (M = 5, F = 6) and those with less than 2 times per week (M = 12, F = 14) shows that more female presented with heartburn. However, there was no statistical difference among the genders with regard to the symptoms recorded. None of these subjects presented with any burning sensation in the mouth or sensitivity in the tongue. In five of those that reported 2 or more weekly history of heartburn, there were minimal losses of tooth structure (TWI scores of 1 and 2 in three and in two subjects respectively) limited to the maxillary central incisors (Table 2).

**Table 1:** Prevalence of GOR and symptoms of GORD

Symptoms	Male (N = 49)	Female (N = 51)	Total
Regurgitation and acidic taste in the mouth	16	19	35
Heartburn ≥ 2 per week	5	6	11
Heartburn per week	7	9	16
Heartburn per month	5	5	10

Chi Square = 0.10; df = 3; p = 0.99.

**Table 2:** Prevalence of dental erosion

	Dental erosion	
Subjects/patients	No (%)	TWI scores
M.O.P. (N = 100)	5 (5)	1 – 2
GORD (N = 125)	20 (16)	1 – 3

Mantel Haenszel chi-square = 5.50, p = 0.02.

One hundred and twenty five patients were diagnosed with GORD. Twenty presented with dental erosion in the maxillary anterior teeth with TWI scores ranging from 1–3. The prevalence of erosion was found to be statistically significant between GORD patients (16%) and controls (5%) (Mantel Haenszel Chi-square 5.50, p < 0.05), Table 2. The result of the gastrointestinal endoscopy reported 41(32.8%) as having reflux oesophagitis, 36 (28.8%) with duodenal ulcer and 48 (38.4%) with gastric ulcer. Eight patients with dental erosion came from the group who had reflux oesophagitis, 7 from the group with duodenal ulcer and 5 from the group with gastric ulcer. Comparison of patients with oesophagitis and those without shows that the mean age of patients with oesophagitis was lower than in those without. The prevalence of erosion was not statistically significant between the endoscopic diagnostic groups (chi-square = 1.33, df = 2, p = 0.51), Table 3.

**Table 3:** Endoscopic diagnostic groups: basic data and number of patients withdental erosion

Diagnostic groups	Number	Mean age (yrs)	Gender		Dental erosion detected
	No (%)	± S.D	Male	Female	
Reflux oesophagitis	41 (32.8)	36.9 ± 9.7	24	17	8
Duodenal ulcer	36 (28.8)	39.2 ± 11.4	16	20	7
Gastric ulcer	48 (38.4)	38.1 ± 11.0	17	31	5

Prevalence of erosion between diagnostic groups: Chi-square = 1.33, df = 2, p = 0.51.

The details of the orodental findings in the twenty patients who had dental erosion are shown in Table 4. There seems to be a slight association between the duration of gastrointestinal symptoms and the severity of erosion. TWI scores of 3 were seen only in those patients whose abdominal symptoms had lasted 10 years or more. The palatal surfaces of the maxillary anterior teeth were usually involved but the central incisors were the most severely affected. Six patients (4 from reflux oesophagitis and 2 from duodenal ulcer) had dental erosion related symptoms in their teeth: sensitivity to cold and heat. The teeth that presented with dentine sensitivity had TWI scores of 3.

**Table 4:** Orodental findings in the twenty patients with GORD who had erosions

S/N	Diagnostic groups***	Age (yrs)	Gender	Duration (yrs)	TWI scores	Dentine Sensitivity**	BMS*
1	RO	26	Female	5	1–2	NP	NP
2	RO	43	Female	10	1–3	P	P
3	RO	44	Male	10	1–3	NP	NP
4	RO	38	Male	5–10	1	NP	NP
5	RO	48	Female	15	2–3	NP	P
6	RO	33	Female	10	1–3	P	P
7	RO	52	Male	15	2–3	P	P
8	RO	50	Female	15	2–3	P	P
9	DU	30	Male	5–10	1–2	NP	NP
10	DU	50	Male	20	2–3	P	P
11	DU	45	Female	15	1–3	NP	NP
12	DU	47	Female	10	1–2	NP	NP
13	DU	33	Female	5	1	NP	NP
14	DU	36	Male	10	1–3	NP	NP
15	DU	43	Male	15	2–3	P	P
16	GU	46	Female	10	1	NP	NP
17	GU	35	Female	10	1–2	NP	NP
18	GU	32	Female	5	1	NP	NP
19	GU	54	Male	20	2–3	NP	P
20	GU	45	Male	15	1–3	NP	P

*BMS = Burning mouth sensation and sensitivity in the tongue

**NP = Not present, P = Present

***RO = Reflux oesophagitis, DU = Duodenal ulcer, GU = Gastric ulcer.

Burning mouth sensation and peppery sensation in the tongue where reported by 9 patients who had had gastrointestinal symptoms for 10–20 years.

## Discussion

Various methods have been employed in the investigation of GOR including endoscopy with biopsy and contrast radiography. Although twenty-four hour oesophageal pH monitoring is considered the gold standard investigation of GOR [20], due to non-availability of the ambulatory pH recorder in our center, GOR was diagnosed by endoscopy where visual identification of mucosal inflammation and oesophagitis was used to identify the existence of GOR. Several other authors have used this method [4,21].

The prevalence of GOR and GORD in the present study was 35% and 11% respectively. The prevalence of GOR was much lower than the 60% reported in the U.K. and other Western societies [13], whereas that of GORD was a little above the reported range of 6%–10% [14,15]. A lower percentage (10%) of the subject studied reported a monthly history of heartburn as compared to 59% of the population reported by Lock *et al *[22]. The present study examined 125 patients with GORD of which 20(16%) had dental erosion. This is also lower than the 24% and 55% reported by Meurman *et al *[16], and Schroeder et al [17] respectively. Although the authors cannot propose any reason for the reported lower prevalence of GOR and GORD, the low prevalence of dental erosion may be related to difference in diet. More so that the thrust of the European studies support the view that erosion (resulting from acidic and carbonated foods and beverages) is more important than attrition in the aetiology of tooth wear [23], whereas, attrition have been reported to be more important than erosion in the aetiology of tooth wear among Nigerians [24,25]. Also in the study of gastro-oesophageal reflux in children and its relationship to erosion of primary and permanent teeth, diet was considered to be a major contributory factor [26].

**Figure 1:**
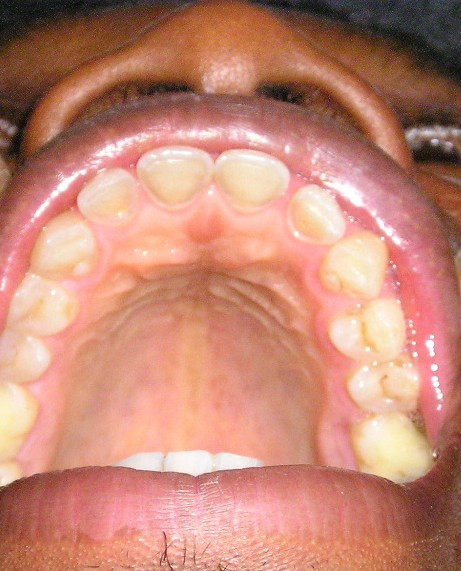
Severe dental erosion affecting the palatal surfaces of the upper anterior teeth in a patient with reflux oesophagitis.

The consumption of acidic beverages among the study population was low, hence the silence on its contribution to the overall disease level. Although this is controversial, the authors are of the opinion that intrinsic (gastric) acid results in palatally eroded sites (as shown in Figure 1) while extrinsic (dietary) acids lead to labial or vestibular erosions. The authors observed some degree of tooth wear (tooth surface loss) on the molar teeth. It was not documented because we believe they are more likely due to "attrition" resulting from rigorous mastication of the more fibrous Nigerian diet. However, acidic refluxate may have been a contributory factor, since tooth surface loss is a multi-factorial disease.

In accordance with the result of Gregory-Head *et al *[27], patients diagnosed with GORD in this study had higher TWI scores compared with control subjects. All the twenty patients that presented with dental erosions in the present study had an underlying gastrointestinal pathosis (Gastric oesophagitis, Duodenal ulcer, and Gastric ulcer) with increased output of acid secretion into the stomach [28]. There was however no statistically significant difference in the prevalence of dental erosions in these diagnostic groupings, Table 3. This is in agreement with the study of Jarvinen *et al *[4]. The severity of dental erosions may depend on the frequency of regurgitation and duration of the gastro-oesophageal reflux. In the present study, patients with TWI scores of 3 had their symptoms for more than 10 years as shown in Table 4. This is supported by the study of Loffeld *et al *[21], which revealed a significant association between duration of complaints and presence of damage in the upper incisors, but in contrast to the findings of Jarvinen *et al *[4] who reported no direct association between the frequency of regurgitation symptoms and the severity of the erosive lesions. Six patients presented with erosion related symptoms in their teeth, they had dentine sensitivity to cold and heat. All of these patients had dental erosions with TWI scores of 3 (Loss of enamel exposing dentine for more than one-third of the surfaces). This probably explains the sensitivity.

Nine patients reported burning mouth sensation and sensitivity in the tongue. They describe the sensation as peppery/burning feeling in the vestibule of the mouth and mostly on the dorsal surface of the tongue. These may have resulted from the prolonged effect of acidic gastric refluxate on the oral mucosa and on the papillae of the tongue.

## Conclusion

The present study supports the consideration of dental erosion as the extra-oesophageal manifestation of GORD. However the association between GORD and burning mouth sensation needs more investigation.

## Competing interests

The author(s) declare that they have no competing interests.

## Authors' contributions

AOO conceived of the study, participated in its design, performed the dental examination including the application of the tooth wear index (TWI), and participated in the initial draft and final write-up of the manuscript. EAA and DAN performed the endoscopic examination of patients with gastro-oesophageal reflux disease, participated in the initial draft and final write-up of the manuscript.

## Pre-publication history

The pre-publication history for this paper can be accessed here:


